# Does the Experience Level of Surgeons and Assistants Influence the Rate of Complications Following Appendicectomy?

**DOI:** 10.7759/cureus.74612

**Published:** 2024-11-27

**Authors:** Joshua Read, Jonathan Johns, Cain Anderson, Jagdish Prasad

**Affiliations:** 1 Ophthalmology, Rotorua Eye Clinic, Rotorua, NZL; 2 General Surgery, Te Whatu Ora, Tauranga, NZL; 3 General Surgery, Te Whatu Ora, Whakatāne, NZL

**Keywords:** appendicectomy, appendicitis, assistant, complication rates, consultant, experience, post operative, registrar, surgeon

## Abstract

Background

Appendicectomies are the most frequently performed acute general surgery. The risk of complications depends on several factors, including patient age, American Society of Anesthesiologists (ASA), duration of symptoms, serum inflammatory markers, and the grade of inflammation. Prior research failed to demonstrate a relationship between the rate of complications and the surgeon’s level of experience. It is unclear if the assistant’s level of experience influences complication rates.

Methods

We conducted a retrospective cohort study to investigate the relationship between the surgeon’s and assistant’s position and the rate of complications following appendicectomy. We also explored whether more experienced staff were involved in higher-risk cases and their relationship with resource utilization.

Results

There was no significant difference in total complication rates based on the surgeon’s and assistant’s position (p = 0.48 and p = 0.99, respectively). Post-operative bleeding was the only complication that correlated with the assistant’s level of experience (p = 0.002). More experienced surgeons performed faster appendicectomies (p =0.002), while the assistant’s position had no influence (p = 0.47). There was no statistically significant relationship between the surgeon’s and assistant’s position, and post-operative length of admission, or risk factors for complications as measured by age, days of abdominal pain, white blood cell count (WCC), C-reactive protein (CRP), ASA, radiographic and intra-operative American Association for the Surgery of Trauma (AAST).

Conclusion

Experienced surgeons performed faster appendicectomies. There was a trend toward higher rates of post-operative bleeding with less experienced assistants. Otherwise, there was no relationship between the surgeon’s experience level and post-appendicectomy complication rates, length of post-operative stay, or patient risk factors for complications.

## Introduction

Appendicectomies are the most common acute general surgery, with a lifetime incidence of 12%-23.1% [1,2]. Their frequency and relative simplicity make them one of the first operations taught to trainees. However, post-operative complications are common. Up to 10.3% of patients develop post-operative wound infections, 9.4% develop intra-abdominal infections, and 0.2% of elderly patients pass away postoperatively [3-6].

Risk factors for complications following appendicectomy include advanced patient age, delayed presentation, higher American Society of Anesthesiologists (ASA) physical status classification, and greater intra-operative inflammation [7-9]. Higher serum white blood cell count (WCC) and C-reactive protein (CRP) are associated with complicated appendicitis [10-12]. Complication rates are also influenced by the surgical approach. Laparoscopic appendicectomies result in fewer wound infections and shorter hospital stays but higher rates of intra-abdominal abscesses [5,13].

Conventional wisdom suggests that more experienced surgeons and assistants would have lower complication rates. This has been demonstrated in a variety of surgical specialties [14-17]. However, this is not a ubiquitous finding, with some research indicating that junior surgeons have lower post-operative complication rates compared to their more experienced counterparts [18-20]. Prior research on appendicectomies failed to demonstrate a relationship between the surgeon’s experience level and the rate of postoperative complications [21-23]. However, the duration of surgery tends to be longer when registrars operate compared to consultants [24]. The influence of the assistant’s level of experience on the rate of post-appendicectomy complications is yet to be examined.

This study aims to investigate whether the surgeon’s and assistant’s level of experience influences the rate of complications following appendicectomy. Secondary aims include examining how staff experience affects surgery duration and length of hospital stay. To investigate for potential confounders, we will explore whether more experienced surgeons and assistants tend to operate on higher-risk cases, as measured by age, ASA classification, WCC and CRP levels at presentation, and American Association for the Surgery of Trauma (AAST) scores.

## Materials and methods

A retrospective cohort study was conducted on patients admitted to Whakatāne Hospital with appendicitis from January 1, 2017, to December 31, 2022. Patients were identified using hospital coding for appendicitis diagnoses. We included all patients admitted for appendicitis who were managed surgically. Patients who were managed non-operatively were excluded from the study.

Participants’ medical records were scrutinized to confirm the diagnosis of appendicitis, determine the presence of risk factors, and determine whether complications occurred. These include the patients’ demographics, ASA, their duration of symptoms, duration of hospital admission, serum WCC, and CRP. Surgery-related post-operative complications were recorded.

Surgical notes were reviewed to determine the type of surgery, surgeon, assistant, and surgery duration. Surgeries were categorized as diagnostic laparoscopy and appendicectomy, laparoscopic appendicectomy, laparoscopic appendicectomy with drain placement, laparoscopic converted to open appendicectomy, and open appendicectomy. Open appendicectomies encompassed laparotomy, gridiron, and Lanz incisions. Both converted and open cases included those with/without drain placement. The operating staff were categorized as consultants, registrars, house surgeons, medical students, or registered nurses. Medical students were in their fourth to sixth years of training. House surgeons are junior doctors, typically in their first or second post-graduate year. Registrars are doctors undergoing advanced specialty training to become fully qualified surgeons. Consultants are surgeons who have completed all specialty training and certification. For the purposes of this analysis, Medical Officers of Specialist Scale (MOSS), experienced doctors who have not completed formal specialty training, were classified alongside registrars. Appendicitis severity was graded radiographically (when applicable) and intra-operatively according to the American Association for the Surgery of Trauma (AAST) severity score [25]. Data was recorded in a password-protected Excel (Microsoft, Redmond, United States) spreadsheet.

Statistical analysis was performed using R (R Foundation for Statistical Computing, Vienna, Austria) software [26]. Ninety-five percent confidence intervals were calculated without the Yates continuity correction when the outcome’s incidence was less than 5%. Discrete variables were analyzed with the Pearson’s Chi-squared test, and pairwise Fisher’s exact test with Bonferroni correction was performed for ad hoc analysis. Continuous variables were analyzed using analysis of variance (ANOVA), and Tukey’s Honest Significant Difference (HSD) test was used for ad hoc analysis. P-values of less than 5% were considered statistically significant. Local study authorization was approved in accordance with the National Ethical Standards for Health and Disability Research and Quality Improvement (study reference 2024-267).

## Results

Table 1 presents the patient demographics and detailed information about the surgeries performed, including the number of procedures and the surgical assistant's position. Columns are grouped according to the surgeon's position. Consultants performed the highest proportion of open appendicectomies. Few appendicectomies were performed by house surgeons. Consultants were most frequently assisted by registrars, while registrars were often assisted by doctors of all positions. Few cases were assisted by medical students and registered nurses.

**Table 1:** Patient demographics and surgical data performed grouped by the surgeon’s position. NZ: New Zealand.

	Consultant N = 133 (41.3%)	Registrar N = 181 (56.2%)	House Surgeon N = 8 (2.5%)	All N = 322
Mean age (years)	33.9	31.1	29.9	32.2
Female	71 (53.4%)	81 (44.8%)	5 (62.5%)	157 (48.8%)
Māori	75 (56.4%	112 (61.9%)	3 (37.5%)	190 (59.0%)
Pasifika	2 (1.5%)	2 (1.1%)	1 (12.5%)	5 (1.6%)
NZ European	46 (34.6%)	55 (30.4%)	3 (37.5%)	104 (32.3%)
Diagnostic laparoscopy and appendicectomy	2 (1.5%)	6 (3.3%)	0	8 (2.5%)
Laparoscopic appendicectomy	95 (71.4%)	144 (79.6%)	8 (100%)	247 (76.7%)
Laparoscopic appendicectomy and drain placement	1 (0.8%)	5 (2.8%)	0	6 (1.9%)
Laparoscopic converted to open appendicectomy	10 (7.5%)	6 (3.3%)	0	16 (5.0%)
Open appendicectomy	25 (18.8%)	20 (11.0%)	0	45 (14.0%)
Assisted by a consultant	1 (0.8%)	23 (12.7%)	0	24 (7.5%)
Assisted by a registrar	98 (73.7%)	58 (32.0%)	8 (100%)	164 (50.9%)
Assisted by a house surgeon	15 (11.3%)	56 (30.9%)	0	71 (22.0%)
Assisted by a medical student	1 (0.8%)	4 (2.2%)	0	5 (1.6%)
Assisted by a registered nurse	2 (1.5%)	3 (1.7%)	0	5 (1.6%)
Assistant not recorded	16 (12.0%)	37 (20.4%)	0	53 (16.5%)

The following two tables show the complication rates after appendicectomy. Table 2 organizes the data by the surgeon’s position, while Table 3 groups it based on the assistant’s position. The medical complications were three cases of hospital-acquired pneumonia and one case each of pleural effusion, pulmonary oedema, atrial fibrillation, bigeminy, acute kidney injury, and electrolyte imbalance.

**Table 2:** The incidence of complications after appendicectomy, grouped by the surgeon’s position.

	Consultant N = 133 (41.3%)	Consultant 95% CI	Registrar N = 181 (56.2%)	Registrar 95% CI	House Surgeon N = 8 (2.5%)	House Surgeon 95% CI	Chi-sq p-value
Any complication	33 (24.8%)	17.9%-33.2%	33 (17.1%)	12.1%-23.6%	0	-	0.09
Wound infection	3 (2.3%)	0.8%-6.4%	5 (2.8%)	1.2%-6.3%	0	-	0.87
Intra-abdominal collection	6 (4.5%)	2.1%-9.5%	11 (6.1%)	3.2%-10.9%	0	-	0.66
Bleeding	3 (2.3%)	0.8%-6.4%	0	-	0	-	0.12
Ileus	7 (5.3%)	2.3%-10.9%	7 (3.9%)	1.9%-7.8%	0	-	0.69
Required reoperation	5 (3.8%)	1.6%-8.5%	5 (3.3%)	1.5%-7.0%	0	-	0.85
Medical complication	6 (4.5%)	2.1%-9.5%	6 (1.7%)	0.6%-4.8%	0	-	0.28

**Table 3:** The incidence of complications after appendicectomy, grouped by the assistant’s position. *Incidence differences across groups reach statistical significance.

	Consultant N = 24 (8.9%)	Consultant 95% CI	Registrar N = 164 (61.0%)	Registrar 95% CI	House Surgeon N = 71 (26.4%)	House Surgeon 95% CI	Medical Student N = 5 (1.9%)	Medical student 95% CI	Registered Nurse N = 5 (1.9%)	Registered Nurse 95% CI	Chi-sq p-value
Any complication	6 (25.0%)	10.6%-47.0%	33 (20.1%)	14.4%-27.2%	15 (21.1%)	14.4%-27.2%	0	-	1 (20.0%)	1.1%-70.1%	0.81
Wound infection	0	-	4 (2.4%)	1.0%-6.1%	2 (2.8%)	0.8%-9.7%	0	-	0	-	0.92
Intra-abdominal Collection	3 (12.5%)	3.3%-33.5%	7 (4.3%)	2.1%-8.5%	4 (5.6%)	1.8%-14.5%	0	-	0	-	0.49
Bleeding	0	-	1 (0.6%)	0.1%-3.4%	1 (1.4%)	0.2%-7.6%	0	-	1 (20.0%)	1.1%-70.1%	0.002*
Ileus	1 (4.2%)	0.7%-20.2%	8 (4.9%)	2.3%-9.7%	4 (5.6%)	1.8%-14.5%	0	-	0	-	0.96
Required reoperation	2 (8.3%)	1.5%-28.5%	4 (2.4%)	1.0%-6.1%	4 (5.6%)	1.8%-14.5%	0	-	0	-	0.51
Medical complication	1 (4.2%)	0.7%-20.2%	5 (3.0%)	1.1%-7.3%	2 (2.8%)	0.8%-9.7%	0	-	0	-	0.98

The only statistically significant difference was observed in bleeding rates when grouped by assistant (p = 0.002). However, post hoc analysis did not demonstrate a statistically significant difference between pairs of groups. The lowest observed p-value in the post hoc analysis was calculated when comparing bleeding rates between the registrar and registered nurse cohorts (0.058). There were no cases of mortality.

The prevalence of risk factors for complications and markers of resource utilization are displayed in the next two tables. Table 4 groups patients by their surgeon’s position and table 5 groups them by their surgical assistant’s position. There were no statistically significant differences between the prevalence of risk factors or the duration of hospital admission among cohorts. ANOVA demonstrated a difference in the length of surgery based on the surgeon’s level of experience (p = 0.026), with ad hoc analysis finding a significant difference between consultants and registrars (p = 0.028). Comparisons between other groups did not reach statistical significance.

**Table 4:** The mean values and 95% confidence intervals of risk factors for complications, grouped by the surgeon’s position. *Incidence differences across groups reaches statistical significance. ANOVA: analysis of variance, WCC: white blood cell count, CRP: C-reactive protein, AAST: American Association for the Surgery of Trauma, ASA: American Society of Anesthesiologists.

	Consultant N = 133 (41.3%)	Registrar N = 181 (56.2%)	House Surgeon N = 8 (2.5%)	ANOVA p-value
Age (years)	33.9 (30.6-37.1)	31.0 (28.4-33.9)	29.9 (20.5-39.2)	0.41
Days of abdominal pain	1.9 (1.6-2.1)	2.0 (1.8-2.2)	1.6 (0.7-2.5)	0.64
WCC (x10^9^ cells per liter)	14.9 (14.1-15.8)	14.8 (14.1-15.5)	13.7 (11.2-16.1)	0.76
CRP (mg/L)	62.9 (48.9-77.0)	57.2 (45.3-69.2)	32.1 (2.7-61.5)	0.52
Radiographic AAST	1.7 (1.6-2.9)	1.7 (1.5-1.9)	1.8 (0-4.1)	0.93
Intraoperative AAST	1.7 (1.5-1.9)	1.6 (1.5-1.8)	1.0 (1.0-1.0)	0.23
Patient ASA	1.3 (1.2-1.4)	1.3 (1.2-1.4)	1.3 (0.9-1.6)	0.85
Surgery duration (minutes)	52.0 (47.9-56.2)	58.8 (55.5-62.1)	63.1 (52.8-73.4)	0.026*
Admission duration (days)	3.1 (2.7-3.6)	2.9 (2.3-3.4)	1.8 (1.2-2.3)	0.47

**Table 5:** The mean values and 95% confidence intervals of risk factors for complications, grouped by the assistant’s position. ANOVA: analysis of variance, WCC: white blood cell count, CRP: C-reactive protein, AAST: American Association for the Surgery of Trauma, ASA: American Society of Anesthesiologists.

	Consultant	Registrar	House Surgeon	Medical Student	Registered Nurse	ANOVA p-value
Age (years)	28.3 (21.3-35.4)	33.5 (30.5-36.5)	32.7 (28.2-37.2)	31.2 (13.6-4.8)	35.8 (12.2-59.4)	0.79
Days of abdominal pain	1.92 (1.3-2.5)	1.91 (1.7-2.1)	1.82 (1.5-2.1)	1.2 (0.6-1.8)	3.2 (0.2-6.2)	0.16
WCC (x10^9^ cells per liter)	14.5 (12.8-16.3)	14.7 (14.0-15.5)	15.2 (14.1-16.3)	12.6 (10.4-14.8)	19.9 (16.0-23.8)	0.12
CRP (mg/L)	56.4 (27.1-85.8)	61.6 (49.2-74.0)	54.9 (33.8-76.1)	58.4 (0-161.4)	64.2 (16.0-123.2)	0.98
Radiographic AAST	1.88 (1.1-2.7)	1.75 (1.5-1.9)	1.47 (1.2-1.8)	1.5 (0-3.1)	1.0 (1.0-1.0)	0.50
Intraoperative AAST	1.91 (1.3-2.5)	1.71 (1.5-1.9)	1.68 (1.4-1.9)	1.4 (0.3-2.5)	1.8 (0.4-3.2)	0.86
Patient ASA	1.21 (1.0-1.4)	1.33 (1.2-1.4)	1.27 (1.1-1.4)	1.4 (0.7-2.1)	1.4 (0.7-2.1)	0.84
Surgery duration (minutes)	59.2 (47.5-71.0)	56.1 (52.6-59.6)	59.6 (54.2-65.0)	53.0 (39.0-67.0)	72.4 (12.9-131.9)	0.47
Admission duration (days)	4.4 (2.6-6.1)	3.0 (2.4-3.5)	3.0 (2.3-3.7)	2.2 (0.8-3.6)	3.6 (0.5-6.7)	0.40

## Discussion

This study found that the assistant experience level influenced post-operative bleeding rates. Additionally, we found that more experienced surgeons perform faster appendicectomies. However, we did not find a difference in the total complication rates or the prevalence of risk factors for complications between groups.

These findings are consistent with the current literature. A meta-analysis by Anyomih et al. also found that consultants were faster than registrars when performing appendicectomies [27]. This may reflect consultants performing more open appendicectomies, which tend to be faster [28]. Similarly, several previous studies also failed to find a difference between the complication rates and length of hospital admission between appendicectomies performed by registrars compared to consultants [21-23]. This may be the result of case allocation, whereby high-risk cases tend to be managed by senior surgeons. In this study, the surgeon selected for each case was determined primarily by availability and competence. Consultants managing more complex cases may explain why they performed a higher proportion of open appendicectomies and were more likely to convert to open. Challenging this theory is the lack of significant differences in the prevalence of risk factors across our cohorts. It is worth noting that the open approach is associated with a higher complication rate, which may offset the advantages of greater surgical experience [29]. Consultants operating on more complex cases may explain their higher proportion of open appendicectomies and conversion to open surgery. However, this interpretation is challenged by the lack of significant differences in the prevalence of risk factors across cohorts. Notably, the open approach is associated with higher complication rates, potentially counterbalancing the benefits of greater surgical experience [29].

This is the first study to investigate how the assistant’s level of experience affects appendicectomy complication rates. Although previous studies have shown that more experienced surgeons have lower complication rates in other types of surgery, such as urological laparoscopic procedures, thoracolumbar osteotomies, and minimally invasive hip arthroplasty [14-17], it remains unclear whether the assistant's experience is also influential in these surgeries. It is possible that assistant experience is more critical in these procedures, although further research is needed to investigate this.

This paper has several key limitations. Firstly, the broad classification of surgeons and assistants may not accurately capture individual levels of experience. For instance, a MOSS may have more surgical experience than a consultant despite being classified as a registrar. Secondly, the limited sample size increases the risk of type II errors. This is especially pertinent for house surgeons who operate it and those assisted by medical students and registered nurses. This critical limitation affects the robustness of our findings regarding less experienced staff. Thirdly, the external validity of our findings regarding consultants is limited. This is because, despite our sample including 12 consultants, just two performed 80% of the consultants’ appendicectomies. Finally, our analysis of appendicitis severity does not encompass every risk factor for complications, for example, those with retrocecal appendicitis with adhesions or morbid obesity.

## Conclusions

The surgeon’s level of experience did not affect the rate of complications following appendicectomy; however, consultants performed faster appendicectomies. These findings suggest that less experienced surgeons can safely perform appendicectomies, although less efficiently. Although assistant experience influenced bleeding rates, ad hoc analysis showed no significant differences between individual groups. There were no other significant differences between complication rates based on the assistant’s level of experience. It is important to note that a registrar and/ or consultant was involved in the operation of every patient. Therefore, we cannot comment on the safety of house surgeons operating in the absence of more experienced colleagues.

## References

[1] D'Souza N (2011). Appendicitis. BMJ Clin Evid.

[2] Addiss DG, Shaffer N, Fowler BS, Tauxe RV (1990). The epidemiology of appendicitis and appendectomy in the United States. Am J Epidemiol.

[3] Gorter RR, Eker HH, Gorter-Stam MA (2016). Diagnosis and management of acute appendicitis. EAES consensus development conference 2015. Surg Endosc.

[4] Bhangu A, Søreide K, Di Saverio S, Assarsson JH, Drake FT (2015). Acute appendicitis: modern understanding of pathogenesis, diagnosis, and management. Lancet.

[5] Jaschinski T, Mosch CG, Eikermann M, Neugebauer EA, Sauerland S (2018). Laparoscopic versus open surgery for suspected appendicitis. Cochrane Database Syst Rev.

[6] Young E, Stewart S, McCulloch GA, Maddern GJ (2019). Appendicectomy mortality: an Australian national audit. ANZ J Surg.

[7] Sohn M, Hoffmann M, Hochrein A, Buhr HJ, Lehmann KS (2015). Laparoscopic appendectomy is safe: influence of appendectomy technique on surgical-site infections and intra-abdominal abscesses. Surg Laparosc Endosc Percutan Tech.

[8] Mouch CA, Cain-Nielsen AH, Hoppe BL (2020). Validation of the American Association for the Surgery of Trauma grading system for acute appendicitis severity. J Trauma Acute Care Surg.

[9] Jiang L, Liu Z, Tong X (2021). Does the time from symptom onset to surgery affect the outcomes of patients with acute appendicitis? A prospective cohort study of 255 patients. Asian J Endosc Surg.

[10] Panagiotopoulou IG, Parashar D, Lin R, Antonowicz S, Wells AD, Bajwa FM, Krijgsman B (2013). The diagnostic value of white cell count, C-reactive protein and bilirubin in acute appendicitis and its complications. Ann R Coll Surg Engl.

[11] Xharra S, Gashi-Luci L, Xharra K, Veselaj F, Bicaj B, Sada F, Krasniqi A (2012). Correlation of serum C-reactive protein, white blood count and neutrophil percentage with histopathology findings in acute appendicitis. World J Emerg Surg.

[12] Koyuncu S, Ismail O (2020). The role of C-reactive protein to lymphocyte ratio in the differentiation of acute and perforated appendicitis. Ulus Travma Acil Cerrahi Derg.

[13] Fleming FJ, Kim MJ, Messing S, Gunzler D, Salloum R, Monson JR (2010). Balancing the risk of postoperative surgical infections: a multivariate analysis of factors associated with laparoscopic appendectomy from the NSQIP database. Ann Surg.

[14] Jerjes W, Hopper C (2018). Surgical experience, workload and learning curve vs postoperative outcome. Eur J Oral Implantol.

[15] Akin Y, Ates M, Celik O, Ucar M, Yucel S, Erdogru T (2013). Complications of urologic laparoscopic surgery: a center surgeon's experience involving 601 procedures including the learning curve. Kaohsiung J Med Sci.

[16] Lau D, Deviren V, Ames CP (2020). The impact of surgeon experience on perioperative complications and operative measures following thoracolumbar 3-column osteotomy for adult spinal deformity: overcoming the learning curve. J Neurosurg Spine.

[17] Hartog YM, Vehmeijer SB (2013). High complication rate in the early experience of minimally invasive total hip arthroplasty by the direct anterior approach. Acta Orthop.

[18] Loderer T, Beretta D, Cozzani F, Bonati E, Rossini M, Del Rio P (2021). Relationship between surgeon experience and adverse events in thyroid surgery. Acta Biomed.

[19] Pasticier G, Timsit MO, Badet L (2006). Nephron-sparing surgery for renal cell carcinoma: detailed analysis of complications over a 15-year period. Eur Urol.

[20] Willeke F, Willeke M, Hinz U (1998). Effect of surgeon expertise on the outcome in primary hyperparathyroidism. Arch Surg.

[21] Barrett JR, Drezdzon MK, Monawer AH, O'Rourke AP, Scarborough JE (2019). Safety in allowing residents to independently perform appendectomy: a retrospective review. J Am Coll Surg.

[22] Chiu CC, Wei PL, Wang W, Chen RJ, Chen TC, Lee WJ, Huang MT (2006). Role of appendectomy in laparoscopic training. J Laparoendosc Adv Surg Tech A.

[23] Wong K, Duncan T, Pearson A (2007). Unsupervised laparoscopic appendicectomy by surgical trainees is safe and time-effective. Asian J Surg.

[24] Mack J, Turner C, Carter D, Hallagan L, Whiting J, Falank C, Sawhney J (2020). The effect of resident participation on appendectomy operative times. J Surg Educ.

[25] Shafi S, Aboutanos M, Brown CV (2014). Measuring anatomic severity of disease in emergency general surgery. J Trauma Acute Care Surg.

[26] R Core Team (2024). R Foundation for Statistical Computing: A language and environment for statistical computing. https://www.R-project.org/.

[27] Anyomih TT, Jennings T, Mehta A (2022). Systematic review and meta-analysis comparing perioperative outcomes of pediatric emergency appendicectomy performed by trainee vs trained surgeon. Pediatr Surg Int.

[28] Katkhouda N, Mason RJ, Towfigh S, Gevorgyan A, Essani R (2005). Laparoscopic versus open appendectomy: a prospective randomized double-blind study. Ann Surg.

[29] Guller U, Hervey S, Purves H, Muhlbaier LH, Peterson ED, Eubanks S, Pietrobon R (2004). Laparoscopic versus open appendectomy: outcomes comparison based on a large administrative database. Ann Surg.

